# ISSEC: inferring contacts among protein secondary structure elements using deep object detection

**DOI:** 10.1186/s12859-020-03793-y

**Published:** 2020-11-05

**Authors:** Qi Zhang, Jianwei Zhu, Fusong Ju, Lupeng Kong, Shiwei Sun, Wei-Mou Zheng, Dongbo Bu

**Affiliations:** 1 Key Lab of Intelligent Information Processing, Big Data Academy, Institute of Computing Technology, Chinese Academy of Sciences, Beijing, 100190 China; 2 School of Computer Science, University of Chinese Academy of Sciences, Beijing, China; 3 Institute of Theoretical Physics, Chinese Academy of Sciences, Beijing, 100190 China

**Keywords:** Protein structure, Secondary structure elements, Inter-SSE contacts

## Abstract

**Background:**

The formation of contacts among protein secondary structure elements (SSEs) is an important step in protein folding as it determines topology of protein tertiary structure; hence, inferring inter-SSE contacts is crucial to protein structure prediction. One of the existing strategies infers inter-SSE contacts directly from the predicted possibilities of inter-residue contacts without any preprocessing, and thus suffers from the excessive noises existing in the predicted inter-residue contacts. Another strategy defines SSEs based on protein secondary structure prediction first, and then judges whether each candidate SSE pair could form contact or not. However, it is difficult to accurately determine boundary of SSEs due to the errors in secondary structure prediction. The incorrectly-deduced SSEs definitely hinder subsequent prediction of the contacts among them.

**Results:**

We here report an accurate approach to infer the inter-SSE contacts (thus called as ISSEC) using the deep object detection technique. The design of ISSEC is based on the observation that, in the inter-residue contact map, the contacting SSEs usually form rectangle regions with characteristic patterns. Therefore, ISSEC infers inter-SSE contacts through detecting such rectangle regions. Unlike the existing approach directly using the predicted probabilities of inter-residue contact, ISSEC applies the deep convolution technique to extract high-level features from the inter-residue contacts. More importantly, ISSEC does not rely on the pre-defined SSEs. Instead, ISSEC enumerates multiple candidate rectangle regions in the predicted inter-residue contact map, and for each region, ISSEC calculates a confidence score to measure whether it has characteristic patterns or not. ISSEC employs greedy strategy to select non-overlapping regions with high confidence score, and finally infers inter-SSE contacts according to these regions.

**Conclusions:**

Comprehensive experimental results suggested that ISSEC outperformed the state-of-the-art approaches in predicting inter-SSE contacts. We further demonstrated the successful applications of ISSEC to improve prediction of both inter-residue contacts and tertiary structure as well.

## Background

Proteins play important roles in a large variety of biological processes. The biological roles of proteins are mainly determined by their three dimensional structures (called *tertiary structures*), making resolving protein structures highly desirable. The experimental technologies to resolve protein tertiary structures, such as X-ray crystallography, NMR spectroscopy, and cryo-electron microscopy, have achieved great successes; however, these technologies are usually time-consuming. Thus, predicting protein structure from amino acid sequence is of great importance [1, 2].

Protein structures are stabilized by both local and global interactions among composing residues; thus, understanding of contacts among residues could effectively facilitate prediction of the correct protein fold [3, 4]. Some recent studies have shown significant improvement in the prediction of inter-residue contacts [5]; however, the application of the predicted residue contact to build protein structure is still far from satisfactory [3, 6, 7]. The potential reasons lie at the errors in the predicted residue-level contacts and the limitations in exploitation of these contacts.

In this study, we focus on the prediction of inter-SSE contacts. The prediction of inter-SSE contacts is of great importance as inter-SSE contacts carry coarse-grain information of tertiary structure and thus could effectively facilitate protein structure prediction [8–10]. In addition, compared with inter-residue contacts, the inter-SSE contacts are much more robust, making it more reliable to predict.

A variety of methods have been developed for the prediction of inter-SSE contacts, which could be roughly divided into two categories, namely, prediction of $\alpha {-}\alpha$ contacts [11–15] and prediction of $\beta {-}\beta$ contacts [16–18]. For example, bbcontacts focuses on the prediction of $\beta {-}\beta$ parallel and antiparallel contacts [7]. It employs the hidden Markov model (HMM) technique to integrate signals covering the predicted inter-residue contacts and the predicted secondary structure. As it directly uses the predicted probability of inter-residue contacts, bbcontacts suffers from the excessive noises existing in these contacts. In contrast to bbcontacts, HHConPred aims to predict $\alpha {-}\alpha$ contacts [19]. It infers the contact between two helices via checking whether these helices show ridge pattern in the inter-residue contact maps. However, as this approach needs pre-defined SSEs, it suffers from the errors of predicted secondary structure. Besides, both of these two approaches were designed for a single type of inter-SSE contacts only and thus could not apply for the proteins with mixed types of inter-SSE contacts.

**Fig. 1:**
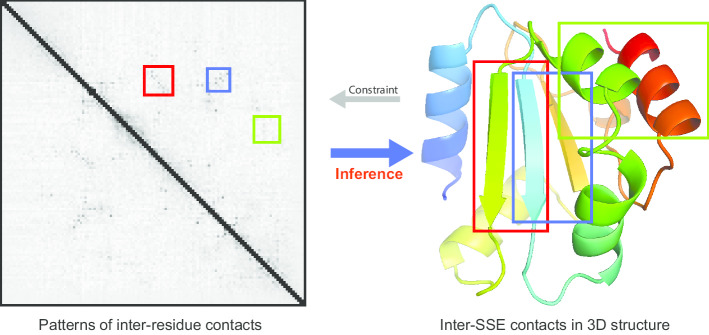
Characteristic patterns formed by contacting SSEs in inter-residue contact map. Two contacting parallel $\beta$-strands often form a diagonal line (rectangle in red), whereas two contacting anti-parallel $\beta$-strands form an anti-diagonal line (rectangle in blue). In contrast, two contacting helices usually form a dashed line (rectangle in green). These characteristic patterns could be used to infer inter-SSE contacts

We here report an accurate approach (called ISSEC) to the prediction of inter-SSE contacts. ISSEC is rooted in the observation that, in the inter-residue contact map, the contacting SSEs usually form a rectangle region with characteristic patterns (Fig. 1 and Additional file 1: Fig. S1). For example, two contacting parallel $\beta$-strands often form a diagonal line, whereas two contacting anti-parallel $\beta$-strands form an anti-diagonal line. In contrast, two contacting helices usually form a dashed line.

ISSEC applies the object detection technique to detect the rectangle regions with characteristic patterns, and infers inter-SSE contacts based on these regions. ISSEC has the following advantages: (1) It uses deep convolution to extract high-level features from the predicted inter-residue contacts and thus could tolerate excessive noises in these contacts. (2) ISSEC examines multiple overlapping regions in the predicted contact map and thus could get rid of the dependency on the pre-defined SSEs. (3) ISSEC could predict multiple types of inter-SSE contact simultaneously. Experimental results on PSICOV [20], CASP11 datasets, and membrane proteins suggest that ISSEC significantly outperformed the existing approaches in prediction accuracy. Furthermore, we successfully applied the predicted inter-SSE contacts to improve the prediction of inter-residue contacts and tertiary structure as well.

## Results

We first explain the concept of ISSEC using protein 3a4tA as a concrete example. Next we show the prediction accuracy of ISSEC on a variety of datasets, including PSICOV dataset, TEST1000 dataset, and transmembrane proteins. Finally, we show the application of ISSEC to improve inter-residue prediction and tertiary structure prediction as well.

### Datasets

From the proteins in PDB25 (Released in February, 2015), we randomly selected 1,000 proteins to construct a test set called TEST1000. The other test sets include: (1) PSICOV118 dataset: consisting of 118 proteins (after excluding 32 proteins without inter-SSE contacts from PSICOV dataset). (2) Mem30 dataset: consisting of 30 transmembrane proteins obtained from MemConP [15]. (3) Mem11 dataset: consisting of 11 transmembrane proteins [19]. (4) BetaSheet186: consisting of 186 proteins that contains $\beta {-}\beta$ contacts [17].

The training set was also constructed based on PDB25. To guarantee low sequence identity between training and testing set, we excluded the proteins with sequence identify over 25% with any protein in the test sets and finally obtained a training set with 9241 proteins.

### The concept of ISSEC using protein 3a4tA as an example

**Fig. 2:**
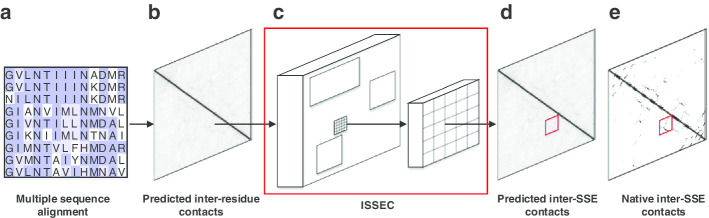
Paradigm of ISSEC. **a** Initially, an multiple sequence alignment (MSA) was built for query protein. **b** The inter-residue contacts were predicted through running CCMPred over the calculated MSA. **c**, **d** ISSEC takes the predicted inter-residue contact map as input and identifies the contacting SSEs (shown as red rectangle). **e** The true inter-SSE contacts (shown as red rectangle) annotated according to native structure of the query protein

The protein 3a4tA is an $\alpha {-}\beta$ protein that consists of 274 residues, forming 20 $\alpha$ and $\beta$ SSEs, i.e., $E_1{-}H_1{-}E_2{-}E_3{-}E_4{-}H_2{-}E_5{-}H_3{-}E_6{-}H_4{-}E_7{-}H_5{-}E_8{-}H_6{-}E_9{-}H_7{-}E_{10}{-}E_{11}{-}E_{12}{-}E_{13}$ (Fig. 2). Here *E* represents a $\beta$ strand and *H* represents an $\alpha$ helix. This protein has a total of 12 inter-SSE contacts, including 2 $\alpha {-}\alpha$ contacts ($H_2{-}H_3$ and $H_6{-}H_7$), 4 $\beta {-}\beta$ parallel contacts ($E_5{-}E_6$, $E_5{-}E_8$, $E_6{-}E_7$, and $E_8{-}E_9$), and 6 $\beta {-}\beta$ anti-parallel contacts ($E_1{-}E_3$, $E_1{-}E_4$, $E_2{-}E_3$, $E_9{-}E_{13}$, $E_{10}{-}E_{13}$ and $E_{12}{-}E_{13}$).

For this protein, ISSEC first predicted probabilities of inter-residue contacts (shown in lower-left triangle of Fig. 2a), which contain a great amount of noises when compared with the true residue contacts (upper-right triangle). Based on these predicted probabilities of inter-residue contacts, ISSEC identified 12 inter-SSE contacts, including 9 positive predictions (rectangle regions in red) and 3 false positives (rectangle regions in green). In addition, 3 inter-SSE contacts ($E_1{-}E_3$, $E_2{-}E_3$ and $H_2{-}H_3$) were missed by ISSEC.

Besides the type of inter-SSE contacts, ISSEC also reports their positions along with confidence scores. Figure 2c shows an example: ISSEC identified an $\alpha {-}\alpha$ contact with confidence score of 0.80. The position of the identified rectangle region matches perfectly with the true position of the contacting SSEs (shown in upper-right triangle). For this rectangle region, ISSEC also reports mask to show the contacting residues. However, the true contacting residues form a dashed line while ISSEC prefers to report a continuous line. This is the reason why ISSEC sets a relatively small weight for the mask loss.

### Accuracy of inter-SSE contact prediction

We first evaluated ISSEC’s performance on proteins that contain single type of inter-SSE contacts. Next, we tested ISSEC on proteins that contain multiple types of inter-SSE contacts.

#### Prediction accuracy of $\alpha {-}\alpha$ contacts for membrane proteins

Most membrane proteins are composed of only $\alpha$-helices. Thus, the accurate prediction of $\alpha {-}\alpha$ contacts should greatly facilitate the prediction of tertiary structure for membrane proteins [21].

We tested ISSEC on Mem30 and Mem11 datasets and performed comparison with three popular approaches, namely, TMhhcp [14], MemConP [15] and HHConPred [19]. Here we followed the convention used in HHConPred to define $\alpha {-}\alpha$ contacts, and comparison criterion from HHConPred [19].

As shown in Table 1, on the Mem30 dataset, TMhhcp and MemConP are able to achieve relatively higher values of precision (61.85% and 70.68%, respectively) but at the substantial sacrifice of recall (15.48% and 24.97%, respectively). This leads to relatively low F-measure for these approaches, thus greatly limiting their applications in membrane proteins. In contrast, HHConPred and ISSEC recalled 44.78% and 57.89% true $\alpha {-}\alpha$ contacts and thus achieved higher F-measure than TMhhcp and MemConP. Furthermore, ISSEC significantly outperformed HHConPred by a large margin (> 6% in F-measure). On the Mem11 dataset, ISSEC outperformed all the three approaches. For example, ISSEC exceeded HHConPred by 13% in precision, 10% in recall, and 3% in F-measure. These results suggested the advantages of ISSEC over the existing approaches.

**Table 1:** Performance of ISSEC, TMhhep, MemConP, and HHConPred on Mem30 and Mem11 datasets

Method	Mem30			Mem11		
	Precision (%)	Recall (%)	F-measure (%)	Precision (%)	Recall (%)	F-measure (%)
TMhhep	61.85	15.48	23.91	36.36	31.60	30.81
MemConP	*70.68*	24.97	34.17	18.18	15.91	16.88
HHConPred	42.92	44.78	41.06	48.67	44.09	39.44
ISSEC	46.44	*57.89*	*47.13*	*61.53*	*54.56*	*43.23*

In each column, the largest element is highlighted in italic. The datasets and the results for all methods except ISSEC were excerpted from [19]

#### Prediction accuracy of $\beta {-}\beta$ contacts on BetaSheet186 dataset

Next, we evaluated ISSEC’s prediction accuracy of the $\beta {-}\beta$ contacts on the BetaSheet186 dataset. Here, we followed the convention used in bbcontacts [7] to define true $\beta {-}\beta$ contacts of proteins.

We compared ISSEC with bbcontacts and summarized the comparison results in Table 2. The table suggested that bbcontacts achieved a higher precision (81.1%) than ISSEC (68.7%) but a lower value of recall (48.2% vs. 66.6%). As results, ISSEC outperformed bbcontacts by 1.6% in terms of F-measure. It is worth pointing out that ISSEC exhibited much higher prediction accuracy of $\beta {-}\beta$ contacts than that of $\alpha {-}\alpha$ contacts (Additional file 1: Fig. S3).

**Table 2:** Performance of ISSEC and bbcontacts on BetaSheet186 dataset

Method	Precision (%)	Recall (%)	F-measure (%)
bbcontacts + PSIPRED	*81.1*	48.2	60.5
ISSEC	68.7	*66.6*	*62.1*

In each column, the largest element is highlighted in italic. The prediction results of bbcontacts were excerpted from [7]

It illustrated the extensiveness of our method that a general model for predicting multiple types of inter-SSE contacts could outperform the tools for single one.

#### Predicting multiple types of inter-SSE contacts simultaneously

To investigate whether ISSEC could predict multiple types of inter-SSE contacts simultaneously, we evaluated ISSEC on TEST1000 and PSICOV118 datasets. Since there had no published tool for the prediction of multiple types of inter-SSE contacts, we just showed the curves on this two datasets. The proteins in these datasets have both $\alpha {-}\alpha$ and $\beta {-}\beta$ contacts. More specifically, the ratio of $\alpha {-}\alpha$, $\beta {-}\beta$ parallel and $\beta {-}\beta$ anti-parallel contacts is 2:1:2 in TEST1000, and 3:2:4 in PSICOV118.

**Fig. 3:**
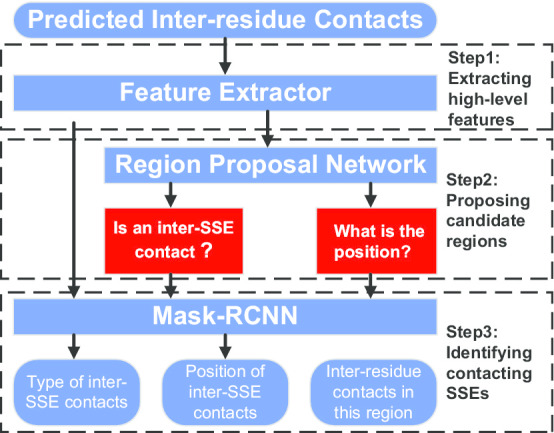
Deep object detection model used by ISSEC. (i) Feature extractor aims to extract high-level features from the predicted probabilities of inter-residue contacts. (ii) Region proposing network aims to propose candidate rectangle regions that might contain contacting SSEs. (iii) Mask-RCNN aims to judge whether a candidate rectangle region contains characteristic patterns or not

As shown in Fig. 3, ISSEC could accurately predict inter-SSE contacts on both PSICOV118 (Precision: 63.24%, Recall: 61.52%, F-measure: 62.36%) and TEST1000 dataset (Precision: 57.22%, Recall: 55.86%, F-measure: 56.53%). The performance on PSICOV118 is marginally better than that on the TEST1000 dataset, which might be rooted in the fact that proteins in PSICOV118 usually have more sequence homologs. In addition, for all of the three types of inter-SSE contacts, the prediction accuracy are considerably close, suggesting that ISSEC could apply on proteins with multiple types of inter-SSE contacts.

### Applying ISSEC to improve prediction of inter-residue contacts

As inter-SSE contacts carry coarse-grain information of structure, it is interesting to examine whether this information could be used to improve prediction of inter-residue contacts. For this aim, we integrated the predicted inter-SSE contacts by ISSEC into the deep residual network model designed for inter-residue contact prediction [5]. The original model was denoted as DeepRN model hereafter.

The original DeepRN model was designed to refine the predicted inter-residue contacts generated using co-evolution technique (e.g., CCMpred [22]). The loss function in the original model is cross entropy summed over all residue pairs. Here we enhanced the original DeepRN model with ISSEC as follows: The residue pairs in the predicted contacting SSEs were assigned with higher weight, i.e., for these residue pairs, their loss are multiplied by $1+S_{t}$, where $S_{t}$ represents the score of predicted inter-SSE contact.

1 $$\begin{aligned} Re\_weighted\ Loss = \sum _{i}\sum _{j} (1+S_{t}) \times CrossEntropy(i,j) \end{aligned}$$

Following the widely-used convention, we divided the contacts into short-, medium- and long-range when the sequence distance of the two contacting residues falls into [6, 11], [12, 23], and $[24, +\infty )$, respectively, and reported the accuracy of top *L*/10, *L*/5, *L*/2, and *L* predicted contacts.

As illustrated in Additional file 1: Tables S2 and S3, when enhanced with ISSEC, DeepRN showed considerable performance improvement in most cases. More importantly as shown in Table 3, the performance improvement are more considerable for long-range contacts on both PSICOV118 dataset (4.2% improvement for top *L* contacts), CASP11 dataset (3.4% improvement for top *L* contacts) and CASP13 dataset (2.0% improvement for top *L*/10 contacts). It was well recognized that the prediction of long-range contacts is a challenging task. This result clearly suggested that the global structure information identified by ISSEC could greatly facilitate accurate prediction of inter-residue contacts.

**Table 3:** Performance improvement of long-range contacts on PSICOV118, CASP11 and CASP13

Method	Long on PSICOV118				Long on CASP11				Long on CASP13			
	*L*/10	*L*/5	*L*/2	*L*	*L*/10	*L*/5	*L*/2	*L*	*L*/10	*L*/5	*L*/2	*L*
CCMpred	0.714	0.591	0.384	0.242	0.472	0.416	0.326	0.244	0.326	0.302	0.218	0.153
DeepRN	0.913	0.879	0.737	0.559	0.723	0.670	0.571	0.440	0.497	0.450	0.339	0.253
DeepRN+ISSEC	*0.919*	*0.892*	*0.777*	*0.601*	*0.730*	*0.686*	*0.599*	*0.474*	*0.517*	*0.451*	*0.350*	*0.269*

### Applying ISSEC to improve 3D structure prediction

When inter-SSE contacts are known, topology of the full tertiary structures are almost fixed. Here, we used the predicted inter-SSE contacts to guide structure building. Specifically, we integrated ISSEC into CONFOLD [23] as follows: CONFOLD consists of two stages, and at the second stage, it identifies strand-pairs from the structures generated at the first stage and uses these pairs to guide structure building. We replaced the second stage of CONFOLD with ISSEC and compared this hybrid version (denoted as CONFOLD+ISSEC) with the original CONFOLD.

**Fig. 4:**
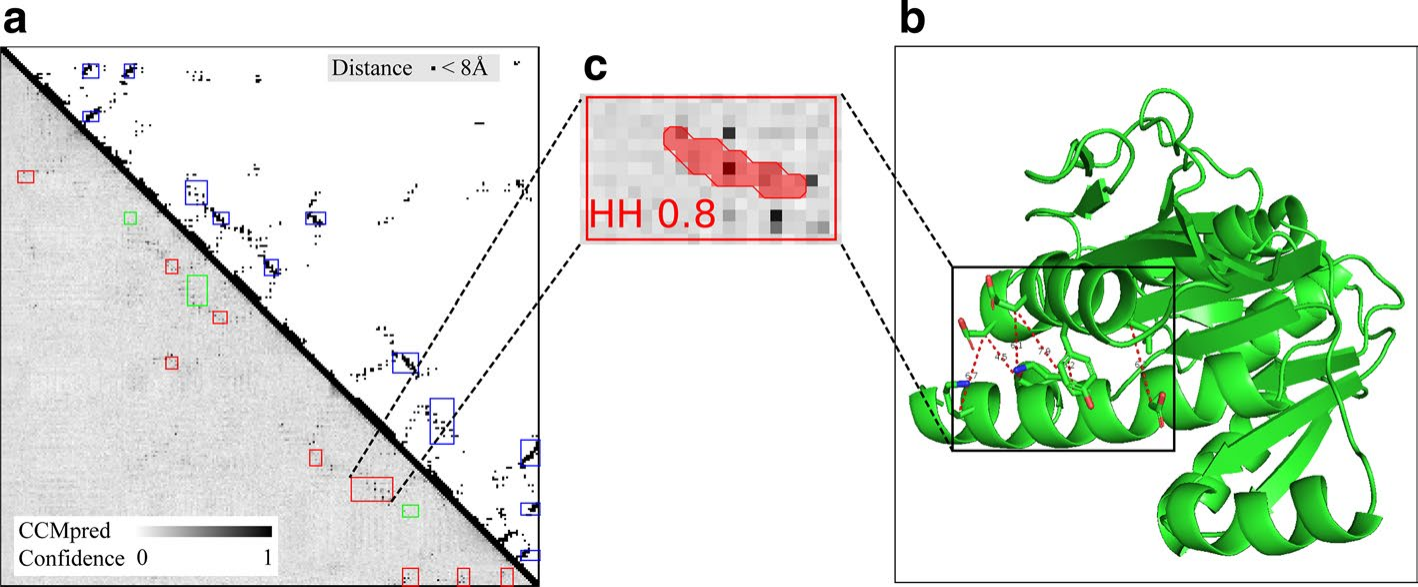
Prediction of inter-SSE contacts for protein 3a4tA using ISSEC. **a** The upper-right triangle shows true inter-residue contacts and true contacting SSEs (rectangle regions in blue). The lower-left triangle shows the predicted contacting SSEs and the predicted probability of inter-residue contacts. Here, rectangle regions in red represent true positive prediction where green ones represent incorrect predictions. **b** The native 3D-structure of the protein 3a4tA. Here, two contacting helices are shown in the box. **c** The prediction results for the contacting helices: type: HH, Confidence score: 0.80, and masks shown in red

**Fig. 5:**
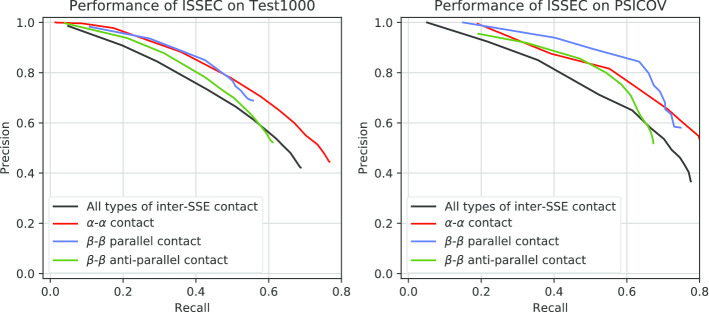
Performance of ISSEC on TEST1000 and PSICOV118 datasets. Black: the performance of all inter-SSE contacts; red: the performance of $\alpha {-}\alpha$ contacts; blue: the performance of $\beta {-}\beta$ parallel contacts; green: $\beta {-}\beta$ anti-parallel contacts

Figure 4 shows the head-to-head comparison of the top model generated by CONFOLD and that by CONFOLD+ISSEC. On 69 out of the 118 proteins in PSICOV118 dataset, CONFOLD+ISSEC generated much better protein structures. We listed 10 of these proteins in Table 4 and exhibited the predicted structures in Fig. 5 and Additional file 1: Figs. S5–S14. Taking protein 1o1zA as an example, CONFOLD generated a structure with TMscore of only 0.44, whereas CONFOLD+ISSEC generated a structure with TMscore of 0.55. For protein 1i4jA and 1ctfA, the TMscore improvement are even higher than 0.20. These results suggested that ISSEC could be used to effectively improve tertiary structure prediction.

**Table 4:** Quality of the predicted structures by using CONFOLD and CONFOLD+ISSEC for ten proteins

Target	CONFOLD		CONFOLD+ISSEC		TM-score
	RMSD	TM-score	RMSD	TM-score	Improvement
1i4jA	13.10	0.27	11.04	0.48	0.21
1ctfA	10.62	0.29	3.90	0.49	0.20
1bdoA	5.97	0.35	4.94	0.47	0.11
1o1zA	10.26	0.44	6.43	0.55	0.11
1rw1A	11.31	0.28	9.28	0.39	0.11
1ktgA	10.56	0.29	7.79	0.40	0.11
1pchA	7.51	0.38	6.05	0.49	0.10
1tqhA	4.43	0.64	3.32	0.73	0.09
1fk5A	15.33	0.26	14.64	0.35	0.09
1npsA	13.54	0.20	11.25	0.28	0.08

## Discussion

Our ISSEC could currently predict three frequent types of inter-SSE contacts, i.e., $\alpha {-}\alpha$, $\beta {-}\beta$ parallel and $\beta {-}\beta$ anti-parallel contacts. How to extend ISSEC to predict $\alpha {-}\beta$ and $\beta$-turn contacts remains one of the future works.

## Conclusions

In this study, we present an approach to predicting inter-SSE contacts. Experimental results suggested that this approach could be used to predict $\alpha {-}\alpha$ contacts for membrane proteins and $\beta {-}\beta$ contacts for $\beta$ proteins. More importantly, it can be used to predict multiple types of inter-SSE contacts simultaneously. Furthermore, our approach could be used to improve prediction of both inter-residue contacts and tertiary structure as well.

## Methods

ISSEC infers inter-SSE contacts through detecting rectangle regions with characteristic patterns in the inter-residue contact map. To detect these rectangle regions, ISSEC employs the object detection framework, which was initially proposed for detecting objects in images [24]. For example, given the image shown in Additional file 1: Fig. S2, the goal is to identify both position and type of the objects (two cats, a dog and a duck in this image).

**Fig. 6:**
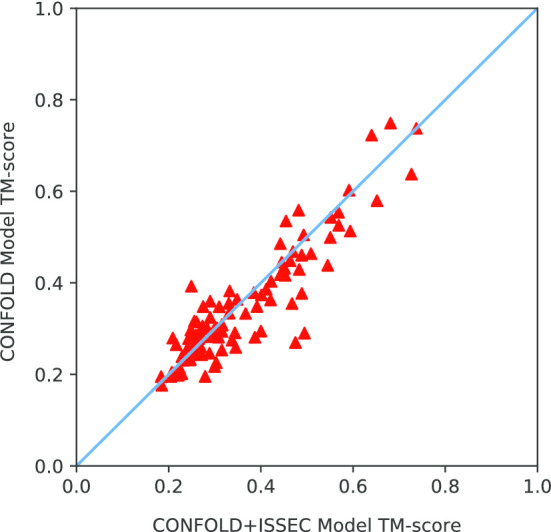
Head-to-head comparison of the structure quality predicted by CONFOLD and CONFOLD+ISSEC. Dataset: PSICOV118

The similarity between inter-residue contact maps and images enables us to apply the object detection technique to infer inter-SSE contacts. Specifically, ISSEC takes the predicted inter-residue contact map as input, and outputs a group of objects representing contacting SSEs (Fig. 6). For each contacting SSE, ISSEC reports its type ($\alpha {-}\alpha$, $\beta {-}\beta$ parallel/anti-parallel contact, further details in Additional file 1), its position (shown as a rectangle), and a confidence score.

**Fig. 7:**
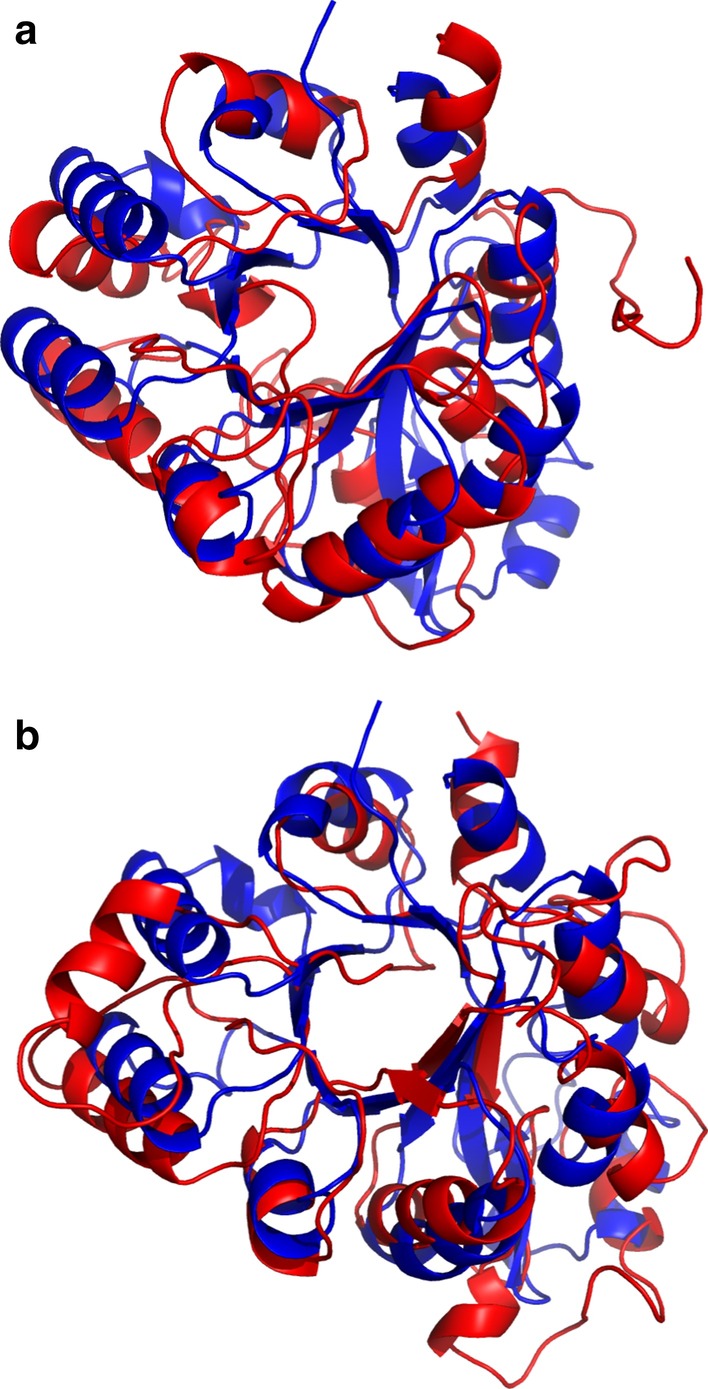
Predicted structure for protein 1o1zA by using CONFOLD (**a**) and CONFOLD+ISSEC (**b**). Here the predicted structures are shown in red whereas the native structure is shown in blue

However, despite the similarities between inter-residue contact maps and images, the contacting SSEs differ greatly from the objects in images. To fit in the specific properties of inter-SSE contacts, we modified the generic object detection framework. Figure 7 shows the main steps of ISSEC, which are described in more details as follows:

1. *Extracting high-level features:* To overcome the drawback of directly using the predicted probabilities of inter-residue contact, ISSEC extracts high-level features using a *feature pyramid network* (FPN, [25]). FPN uses both bottom-up and top-down pathways linked through lateral connections, enabling it to generate a collections of feature maps at multiple levels for subsequent analysis.
2. *Proposing candidate rectangle regions:* As we have no knowledge of location or size of the rectangle regions formed by contacting SSEs in advance, we propose multiple candidate rectangle regions and expect at least one of these proposed regions to cover the contacting SSEs. ISSEC accomplishes this objective using a *region proposal network* (RPN, [26]), which operates in two steps: (1) *Generating seed rectangle region on feature maps:* ISSEC scans the generated feature maps and generates 9 rectangle regions circled at each element on feature maps. (2) *Tracing back to the input inter-residue contact map:* From each rectangle region on the feature map, ISSEC traces back to a larger rectangle region on the input inter-residue contact map. This tracing back operation is accomplished using ROIAlign [27].
  It is worthy pointing out that the rectangle regions formed by contacting SSEs are relatively small; more specifically, each of these regions does not exceed 1/4 of the input inter-residue contact map. This observation provides the possibility to cover all contacting SSEs using the proposed candidate rectangle regions. ISSEC achieves this objective through appropriately choosing various shape and size of the rectangle regions on the feature maps (see Additional file 1: Table S1 for details).
3. *Identifying contacting SSEs:* For each of the proposed candidate rectangle regions, ISSEC calculates a confidence score to measure whether this region contains characteristic patterns of inter-SSEs or not. Based on the characteristic pattern, ISSEC also determines the type of inter-SSEs contacts, i.e., $\alpha {-}\alpha$, $\beta {-}\beta$ parallel or anti-parallel. In addition, since the candidate rectangle region might be larger than the true size of contacting SSEs, a calibrating operation is also needed to shrink the rectangle region and calculate the true position of the contacting SSEs as well. ISSEC employs the Mask-RCNN [27] technique to achieve these objectives.
  Unlike the task of detecting objects in images, the output of Mask-RCNN cannot be directly used to infer contacting SSEs. The reasons are rooted in the difference between the objects in images and contacting SSEs: (1) as shown in Additional file 1: Fig. S2, two objects in images, say the cat and the dog, overlap significantly. In contrast, two rectangle regions formed by contacting SSEs never overlap. (2) Moreover, a rectangle region formed by contacting SSEs would not overlap with diagonal line.
  To identify non-overlapping rectangle regions, ISSEC uses a *greedy selection* strategy that works as follows: We first filter out the rectangle regions that overlap with diagonal and the regions whose confidence score less than a threshold ($T=0.70$ in this study). Next, we sort the remainder in the decreasing order of their confidence score. Then, we select the top rectangle region of the remainder and remove any rectangle regions that overlap with the selected one. This *selecting and removing* step is repeated until all candidate rectangle regions were processed.
  For each of the selected non-overlapping rectangle regions, ISSEC reports the corresponding inter-SSE contact type, position, and confidence score as final results. Finally, a rectangle region that had > 80% overlap with a native contact was correct one.

### Loss function design

In the training process, ISSEC uses the multi-task loss, including classification loss, localization loss and mask loss (further details in Additional file 1). Briefly speaking, the classification loss measures the difference between the predicted and true types of contacting SSEs. The localization loss measures the difference between the position of rectangle region and the true position of SSEs. The localization loss enables ISSEC to acquire accurate position of SSEs, thus avoiding the needs of pre-defined SSE boundaries.

Unlike the $\beta {-}\beta$ parallel and anti-parallel contacts, the $\alpha {-}\alpha$ contacts appear as dashed lines, which is unsuitable for the mask mechanism. In this study, we set the weight of mask loss smaller than those of the classification and localization losses.

## Supplementary information

**Additional file 1:** Supplementary methods, tables and figures.

## Data Availability

The datasets generated and/or analysed during the current study, together with the codes are available via https://github.com/bigict/ISSEC/.

## Abbreviations

ISSEC: Inferring secondary structure element contact
SSE: Secondary structure element
NMR: Nuclear magnetic resonance
HMM: Hidden Markov model
RPN: Region proposal network

## References

[1] Branden CI (1999). Introduction to protein structure.

[2] Floudas CA (2007). Computational methods in protein structure prediction. Biotechnol Bioeng.

[3] Kim DE, DiMaio F, Yu-Ruei Wang R, Song Y, Baker D (2014). One contact for every twelve residues allows robust and accurate topology-level protein structure modeling. Proteins Struct Funct Bioinform.

[4] Zhu J, Zhang H, Li SC, Wang C, Kong L, Sun S, Zheng W-M, Bu D (2017). Improving protein fold recognition by extracting fold-specific features from predicted residue–residue contacts. Bioinformatics.

[5] Wang S, Sun S, Li Z, Zhang R, Xu J (2017). Accurate de novo prediction of protein contact map by ultra-deep learning model. PLoS Comput Biol.

[6] Skolnick J, Kolinski A, Ortiz AR (1997). MONSSTER: a method for folding globular proteins with a small number of distance restraints 1. J Mol Biol.

[7] Andreani J, Söding J (2015). Bbcontacts: prediction of *β*-strand pairing from direct coupling patterns. Bioinformatics.

[8] Barth P, Schonbrun J, Baker D (2007). Toward high-resolution prediction and design of transmembrane helical protein structures. Proc Natl Acad Sci.

[9] Eilers M, Patel AB, Liu W, Smith SO (2002). Comparison of helix interactions in membrane and soluble *α*-bundle proteins. Biophys J.

[10] Ruczinski I, Kooperberg C, Bonneau R, Baker D (2002). Distributions of beta sheets in proteins with application to structure prediction. Proteins Struct Funct Bioinform.

[11] Lo A, Chiu Y-Y, Rødland EA, Lyu P-C, Sung T-Y, Hsu W-L (2009). Predicting helix–helix interactions from residue contacts in membrane proteins. Bioinformatics.

[12] Fuchs A, Kirschner A, Frishman D (2009). Prediction of helix–helix contacts and interacting helices in polytopic membrane proteins using neural networks. Proteins Struct Funct Bioinform.

[13] Yang J, Jang R, Zhang Y, Shen H-B (2013). High-accuracy prediction of transmembrane inter-helix contacts and application to GPCR 3D structure modeling. Bioinformatics.

[14] Wang X-F, Chen Z, Wang C, Yan R-X, Zhang Z, Song J (2011). Predicting residue–residue contacts and helix–helix interactions in transmembrane proteins using an integrative feature-based random forest approach. PLoS ONE.

[15] Hönigschmid P, Frishman D (2016). Accurate prediction of helix interactions and residue contacts in membrane proteins. J Struct Biol.

[16] Baldi P, Pollastri G, Andersen CA, Brunak S. Matching protein beta-sheet partners by feedforward and recurrent neural networks. In: Proceedings of the 2000 conference on intelligent systems for molecular biology (ISMB00), La Jolla: AAAI Press; 2000, p. 25–36. 10977063

[17] Cheng J, Baldi P (2005). Three-stage prediction of protein *β*-sheets by neural networks, alignments and graph algorithms. Bioinformatics.

[18] Savojardo C, Fariselli P, Martelli PL, Casadio R (2013). BCov: a method for predicting *β*-sheet topology using sparse inverse covariance estimation and integer programming. Bioinformatics.

[19] Xiong D, Mao W, Gong H (2017). Predicting the helix–helix interactions from correlated residue mutations. Proteins Struct Funct Bioinform.

[20] Jones DT, Buchan DW, Cozzetto D, Pontil M (2011). PSICOV: precise structural contact prediction using sparse inverse covariance estimation on large multiple sequence alignments. Bioinformatics.

[21] Hildebrand PW, Lorenzen S, Goede A, Preissner R (2006). Analysis and prediction of helix–helix interactions in membrane channels and transporters. Proteins Struct Funct Bioinform.

[22] Seemayer S, Gruber M, Söding J (2014). CCMpred: fast and precise prediction of protein residue–residue contacts from correlated mutations. Bioinformatics.

[23] Adhikari B, Bhattacharya D, Cao R, Cheng J (2015). CONFOLD: residue–residue contact-guided ab initio protein folding. Proteins Struct Funct Bioinform.

[24] Redmon J, Divvala S, Girshick R, Farhadi A. You only look once: unified, real-time object detection. In: Proceedings of the IEEE conference on computer vision and pattern recognition; 2016, pp. 779–88.

[25] Lin T-Y Dollá P, Girshick RB, He K, Hariharan B, Belongie SJ. Feature pyramid networks for object detection. In: The IEEE conference on computer vision and pattern recognition (CVPR); 2017.

[26] Ren S, He K, Girshick R, Sun J. Faster R-CNN: towards real-time object detection with region proposal networks. In: Advances in neural information processing systems; 2015, pp. 91–9. 10.1109/TPAMI.2016.2577031 27295650

[27] He K, Gkioxari G, Dollár P, Girshick R. Mask R-CNN. In: 2017 IEEE international conference on computer vision (ICCV). New York: IEEE; 2017, pp. 2980–8.

