# Supplementary material for: ISSEC: inferring contacts among protein secondary structure elements using deep object detection
Source: BMC Bioinformatics. 2020 Nov 5;21:503. doi: 10.1186/s12859-020-03793-y (PMC7643357; doi:10.1186/s12859-020-03793-y)
Supplement: Supplementary file 1 — Additional file 1: Supplementary methods, tables and figures. [file 12859_2020_3793_MOESM1_ESM.pdf]

1. Supplementary Methods

1.1. Input of the ISSEC

ISSEC's input includes the predicted probabilities of inter-residue contacts generated by CCMpred and 3-state secondary structure generated by PSIPRED [3]. The 3-state secondary structure was transformed into a matrix as follows: For a pair of residues i and j, we concatenate v_i and v_j into a single vector, which is used as input feature of this residue pair. Here, v_i refers to the probability distributions of 3-state secondary structure of residue i. Thus, we obtained a $L \times L \times 7$ feature map as input.

1.2. Definition of inter-SSE contacts

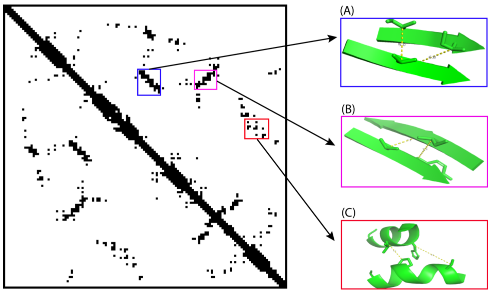

Figure 1: Inter-SSE contacts (right panel) and corresponding characteristic patterns in inter-residue contact map (left panel) for protein 1a1t. (A) a regular β-β parallel contact between two β-strands, (B) a regular β-β anti-parallel contact between two β-strands, (C) an α-α contact between two α helices.

In the study, we focused on 3 types of inter-SSE contacts, namely, α-α contact, β-β parallel contact, and β-β anti-parallel contact (Supplementary Figure 1). The definitions of these three classes are listed below:

- *α-α contact*: A pair of helices H_1 and H_2 is defined as contact if there is a residue-residue contact between them. The helices should have at least 6 residues.
- *Parallel (anti-parallel) β-β contact*: We adopted the definition used by bbcontacts [1], i.e., a continuous diagonal (sub-diagonal) in the inter-residue contact map is defined as β-β parallel (anti-parallel) contact.

1.3. Loss Function and Implementation Details

ISSEC uses multi-task loss function [4, 2] that combines the loss of classification, localization and segmentation mask, i.e.,

$$L = L_{class} + L_{bbox} + L_{mask} \tag{1}$$

- L_{class} represents the loss function over 4 classes (α-α contact, β-β parallel contact, β-β anti-parallel contact, and background).

$$L_{class} = \frac{1}{4}\sum_{i=1}^{4}[-p_i^* \log p_i - (1 - p_i^*)\log(1 - p_i)] \tag{2}$$

where p_i denotes the probability that the rectangle region of interest represents the i-th type of inter-SSE contact, and p_i^* represents the ground-truth.

- L_{bbox} measures the difference between the predicted position and true position of the inter-SSE contact.

$$L_{bbox} = p_i^* \cdot \sum_{i \in \{x,y,w,h\}} L_1^{\text{smooth}} (b_i - b_i^*) \tag{3}$$

$$L_1^{\text{smooth}}(x) = \begin{cases} 0.5x^2 & \text{if } |x| < 1 \\ |x| - 0.5 & \text{otherwise} \end{cases} \tag{4}$$

where b_i represents one of four points of predicted bounding box, while b_i^* represents its groundtruth. Here, L_1^{smooth} is the smooth L1-norm loss. Using the L_{bbox} term, ISSEC could shrink the rectangle region and calculate the true position of the contacting SSEs.

- L_{mask} is defined using the average binary cross-entropy loss as follows:

$$L_{mask} = -\frac{1}{m^2} \sum_{1 \leq i,j \leq \ m} [y_{ij} \log \ \hat{y}_{ij}^k + (1 - y_{ij}) \log(1 - \hat{y}_{ij}^k)] \tag{5}$$

The rectangle region of interest was first transformed into 4 $m \times m$ matrices using ROIAlign, where each matrix represent mask of a type of inter-SSE contact (α-α contact, β-β parallel contact, β-β anti-parallel contact, and background). For each element (i, j) in the matrix, y_{ij} denotes the true mask whereas $\hat{y}_{ij}^k$ denotes the predicted mask for type k.

Hyper Parameters: ResNet-50 was used as backbone to extract image features, all conversion filter were 3 x 3, except the output conversion filter were 1 x 1, we used ReLU in hidden layers.

Model Implementation and Efficiency: The optimization used the momentum optimizer with learning rate 0.002, with L2-norm regularization to prevent overfitting, and early stopping with max iteration of 100000. The whole algorithm is implemented using TensorFlow on a NVIDIA 1070 GPU, the batch size is set to 2 due to GPU limitation, and the training time is less than 15 hours.

2. Supplementary Tables and Figures

Table 1: Shapes and sizes of proposed rectangle regions in the feature maps. Here the shape of a rectangle is described using the ratio of length to width.

No	Ratio of length to width	Size (in pixel)
1	0.5	16
2	0.5	32
3	0.5	64
4	1.0	16
5	1.0	32
6	1.0	64
7	2.0	16
8	2.0	32
9	2.0	64

Table 2: Performance of CCMpred, DeepRN, and DeepRN+ISSEC on PSICOV118 dataset

Method	Short				Medium				Long			
	$L/10$	$L/5$	$L/2$	L	$L/10$	$L/5$	$L/2$	L	$L/10$	$L/5$	$L/2$	L
CCMpred	0.468	0.318	0.178	0.120	0.540	0.375	0.198	0.123	0.714	0.591	0.384	0.242
DeepRN	0.871	0.749	0.471	0.282	0.842	0.735	0.508	0.324	0.913	0.879	0.737	0.559
DeepRN+ISSEC	**0.877**	**0.766**	**0.488**	**0.288**	**0.864**	**0.758**	**0.536**	**0.334**	**0.919**	**0.892**	**0.777**	**0.601**

Table 3: Performance of CCMpred, DeepRN, and DeepRN+ISSEC on CASP11 dataset

Method	Short				Medium				Long			
	$L/10$	$L/5$	$L/2$	L	$L/10$	$L/5$	$L/2$	L	$L/10$	$L/5$	$L/2$	L
CCMpred	0.378	0.293	0.187	0.128	0.426	0.334	0.220	0.153	0.472	0.416	0.326	0.244
DeepRN	0.755	0.624	0.400	0.251	**0.758**	0.668	0.479	0.312	0.723	0.670	0.571	0.440
DeepRN+ISSEC	**0.759**	**0.642**	**0.411**	**0.252**	0.746	**0.678**	**0.483**	**0.317**	**0.730**	**0.686**	**0.599**	**0.474**

Table 4: Performance of CCMpred, DeepRN, and DeepRN+ISSEC on CASP13 dataset

Method	Short				Medium				Long			
	$L/10$	$L/5$	$L/2$	L	$L/10$	$L/5$	$L/2$	L	$L/10$	$L/5$	$L/2$	L
CCMpred	0.304	0.253	0.170	0.138	0.347	0.277	0.190	0.141	0.326	0.302	0.218	0.153
DeepRN	**0.543**	0.472	0.370	0.262	0.558	**0.506**	0.370	**0.272**	0.497	0.450	0.339	0.253
DeepRN+ISSEC	0.534	**0.505**	**0.371**	**0.262**	**0.584**	0.491	**0.372**	0.263	**0.517**	**0.451**	**0.350**	**0.269**

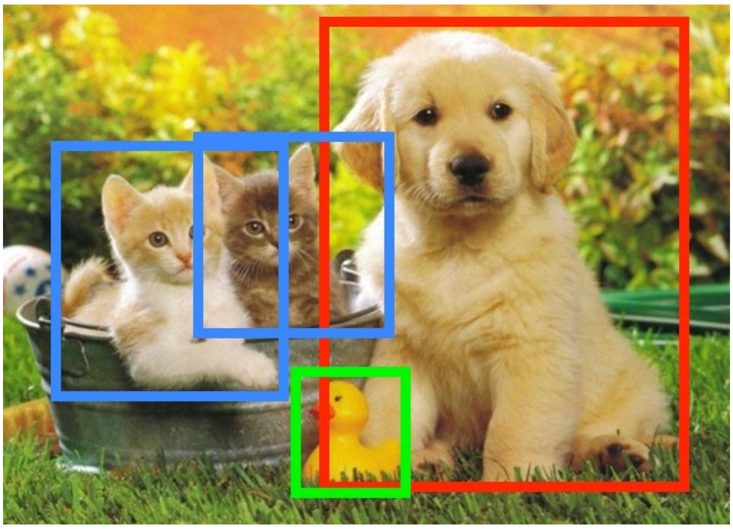

Figure 2: An example of object detection in image. This image contains two cats, a dog and a duck, and these objects overlap considerably.

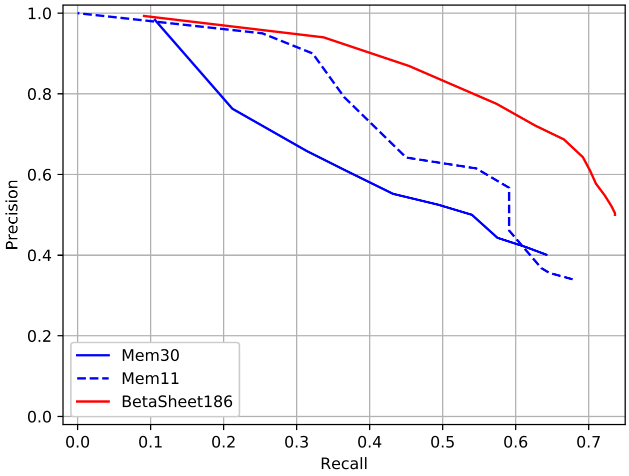

Figure 3: Performance of ISSEC on Mem30, Mem11 and BetaSheet186 dataset.

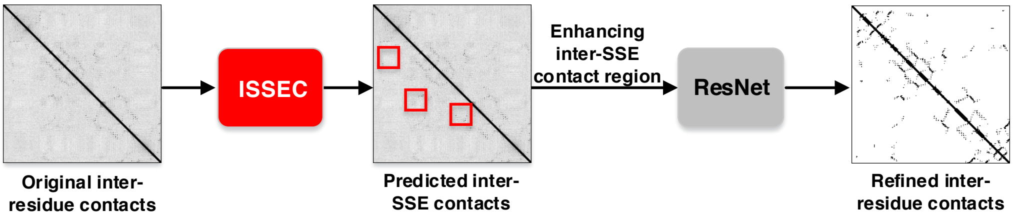

Figure 4: Improving inter-residue contacts by re-weighting the loss function of Xu's model based the predicted inter-SSE contacts by our ISSEC. In Xu's model, the CCMpred was fed into the ResNet, and then a improved inter-residue contact was predicted. In our re-weighting strategy, the CCMpred was also fed into ISSEC to predicted inter-SSE contacts (Red boxes), which were then used to re-weighting loss function of Xu's model.

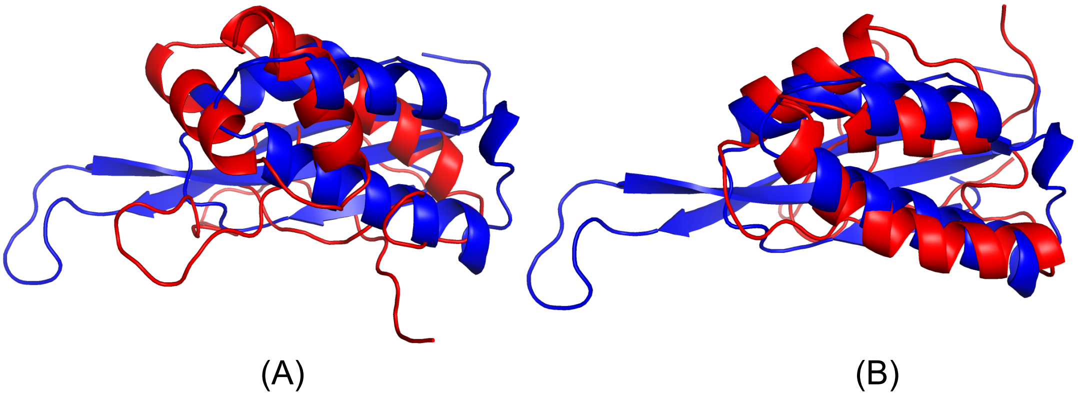

Figure 5: Predicted structure for protein `1i4jA` by using CONFOLD (A) and CONFOLD+ISSEC (B). Here the predicted structures are shown in red whereas the native structure is shown in blue.

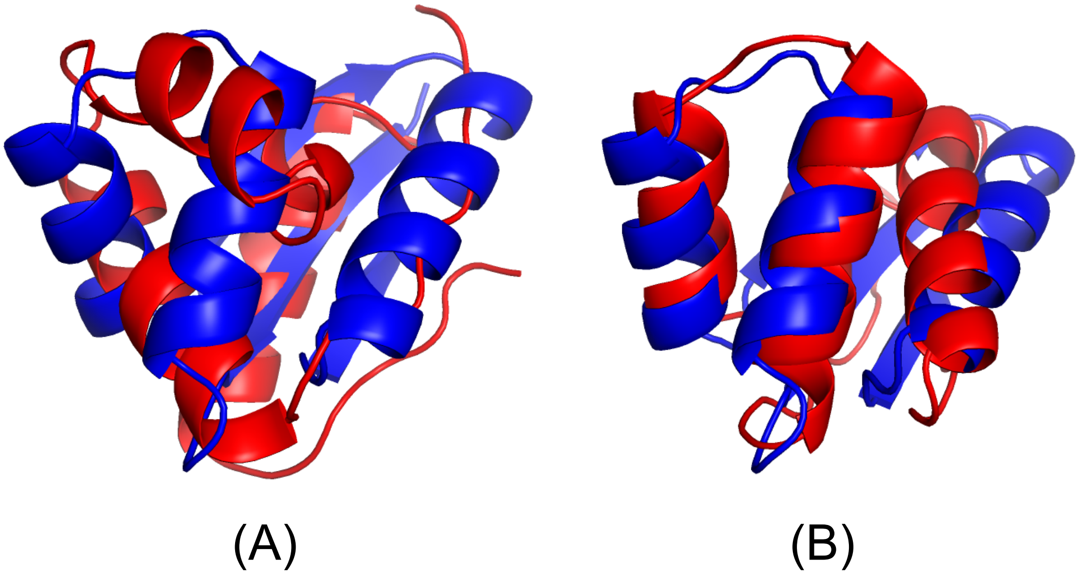

Figure 6: Predicted structure for protein `1ctfA` by using CONFOLD (A) and CONFOLD+ISSEC (B). Here the predicted structures are shown in red whereas the native structure is shown in blue.

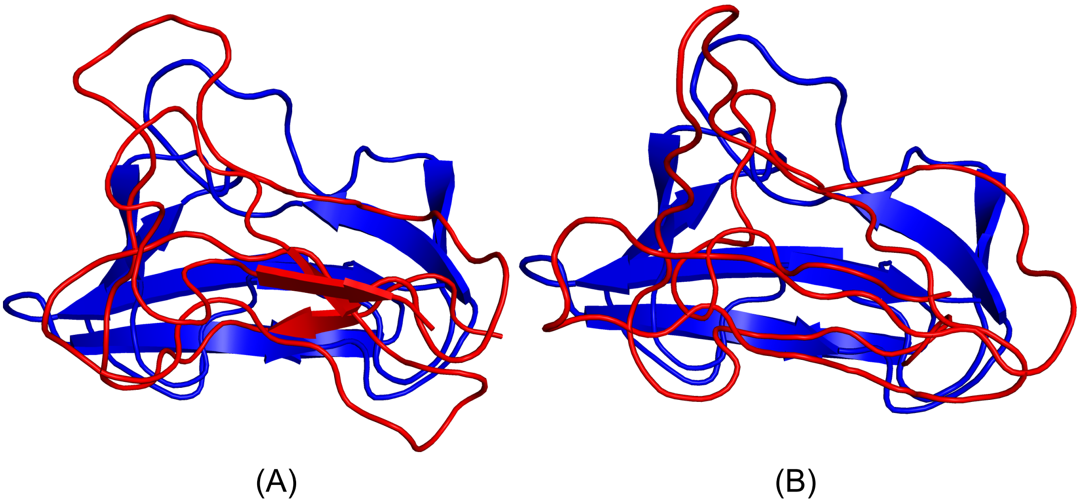

Figure 7: Predicted structure for protein `1bdoA` by using CONFOLD (A) and CONFOLD+ISSEC (B). Here the predicted structures are shown in red whereas the native structure is shown in blue.

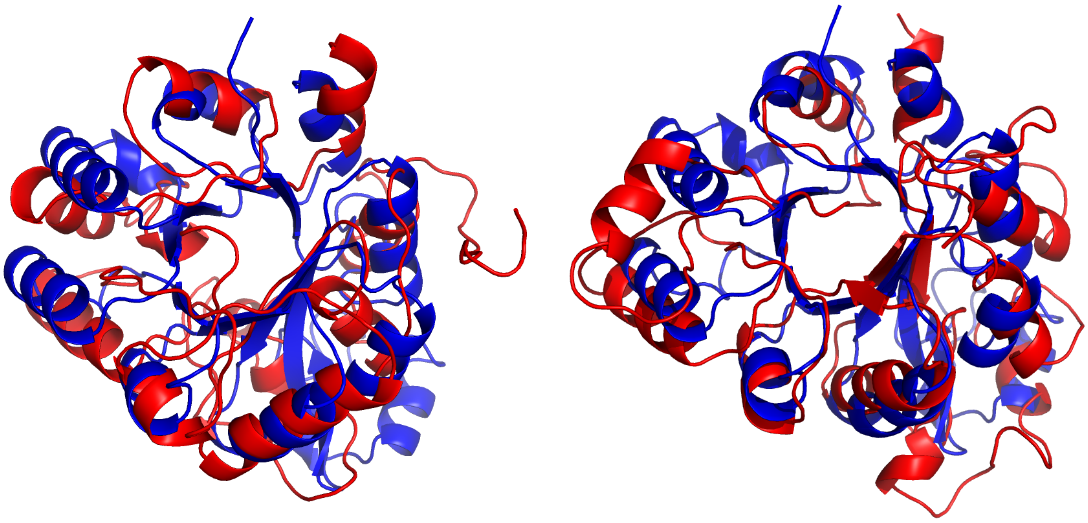

(A) (B)

Figure 8: Predicted structure for protein `1o1zA` by using CONFOLD (A) and CONFOLD+ISSEC (B). Here the predicted structures are shown in red whereas the native structure is shown in blue.

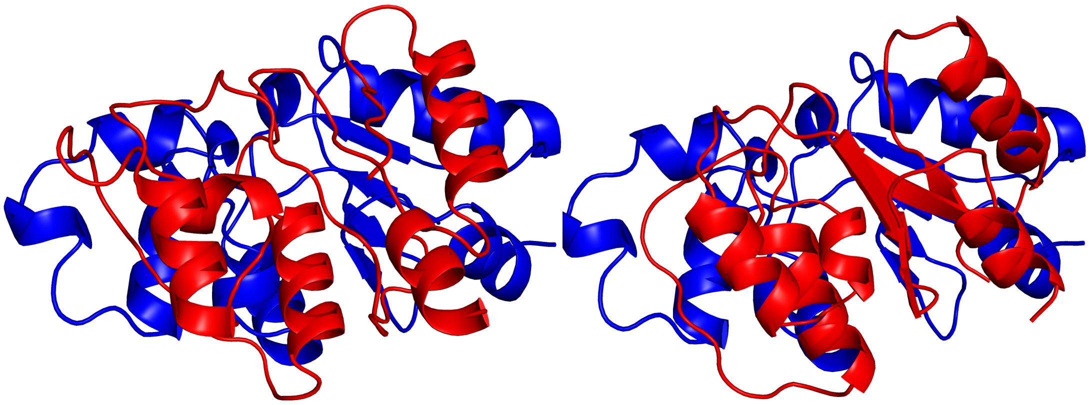

(A) (B)

Figure 9: Predicted structure for protein `1rw1A` by using CONFOLD (A) and CONFOLD+ISSEC (B). Here the predicted structures are shown in red whereas the native structure is shown in blue.

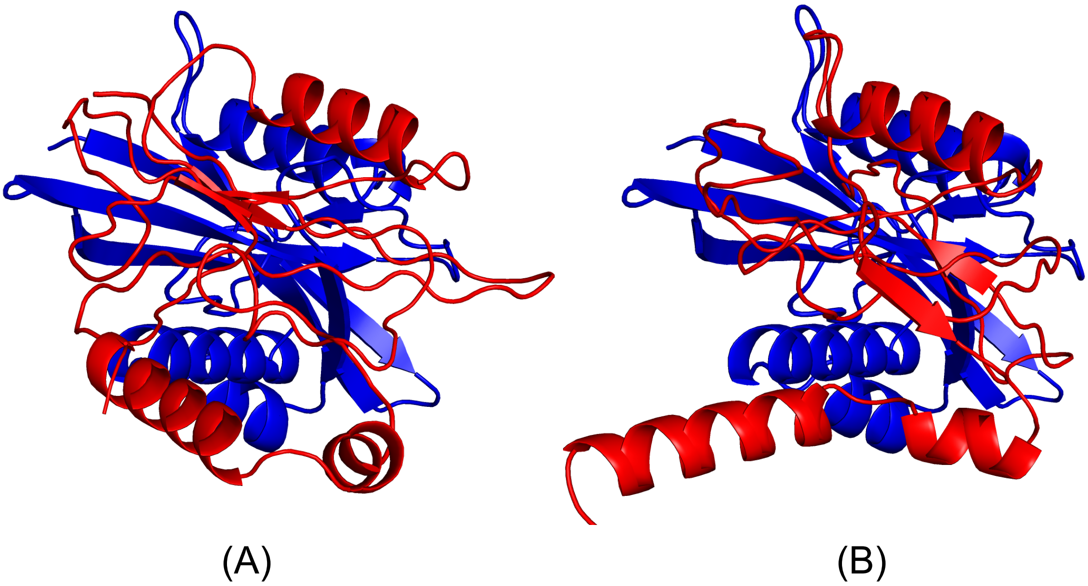

Figure 10: Predicted structure for protein `1ktgA` by using CONFOLD (A) and CONFOLD+ISSEC (B). Here the predicted structures are shown in red whereas the native structure is shown in blue.

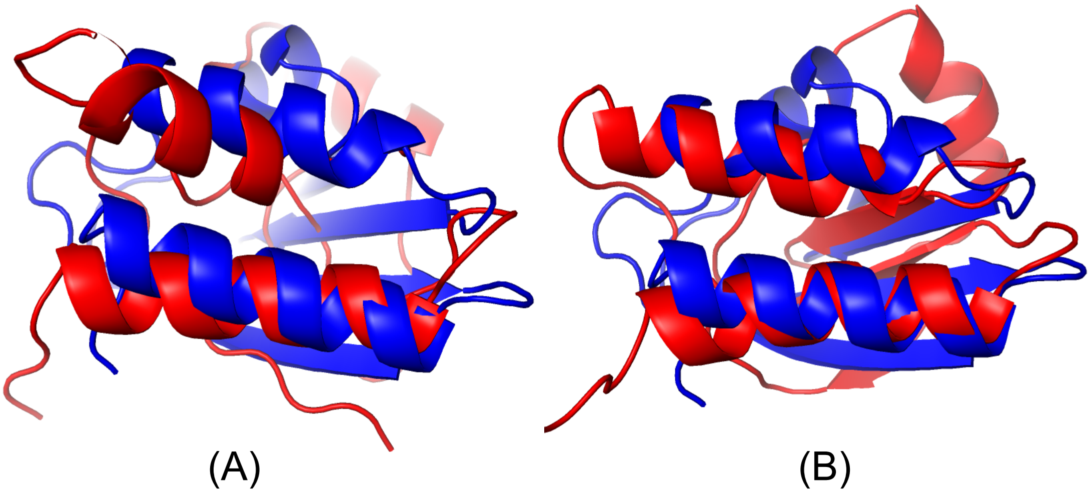

Figure 11: Predicted structure for protein `1pchA` by using CONFOLD (A) and CONFOLD+ISSEC (B). Here the predicted structures are shown in red whereas the native structure is shown in blue.

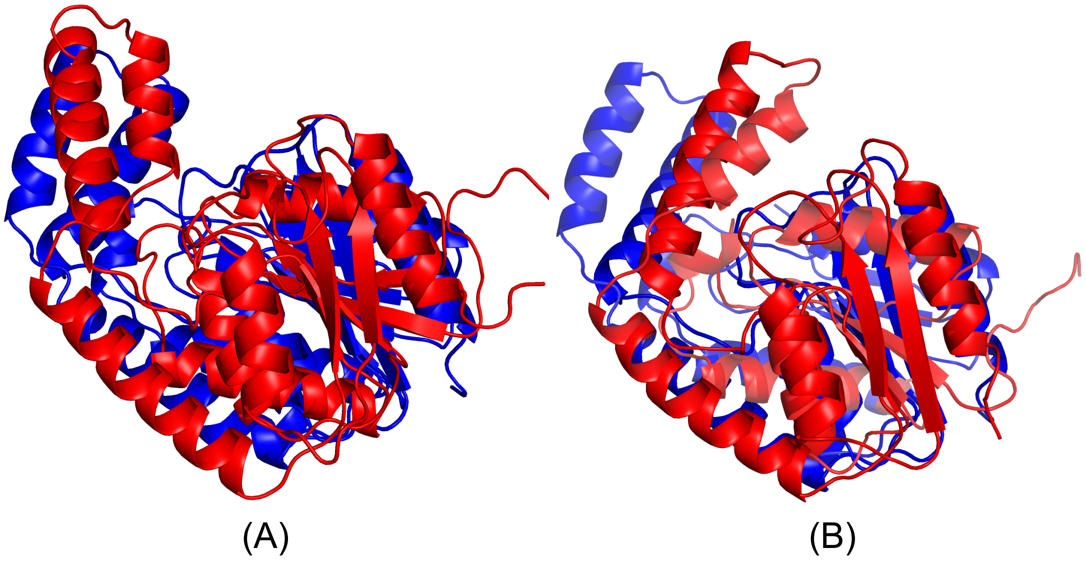

Figure 12: Predicted structure for protein `1tqhA` by using CONFOLD (A) and CONFOLD+ISSEC (B). Here the predicted structures are shown in red whereas the native structure is shown in blue.

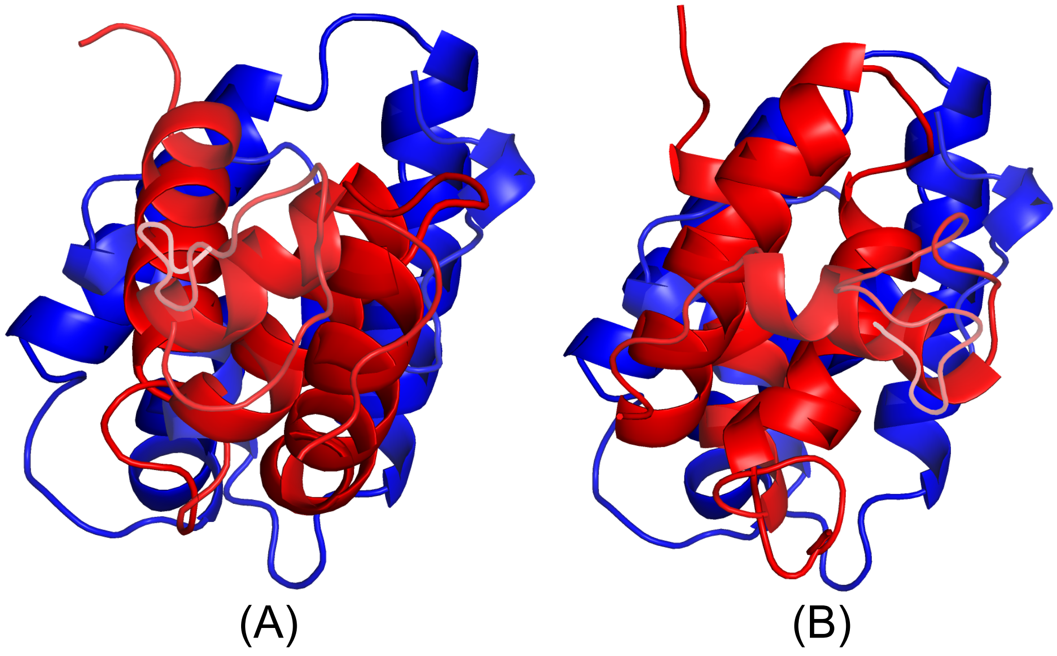

Figure 13: Predicted structure for protein `1fk5A` by using CONFOLD (A) and CONFOLD+ISSEC (B). Here the predicted structures are shown in red whereas the native structure is shown in blue.

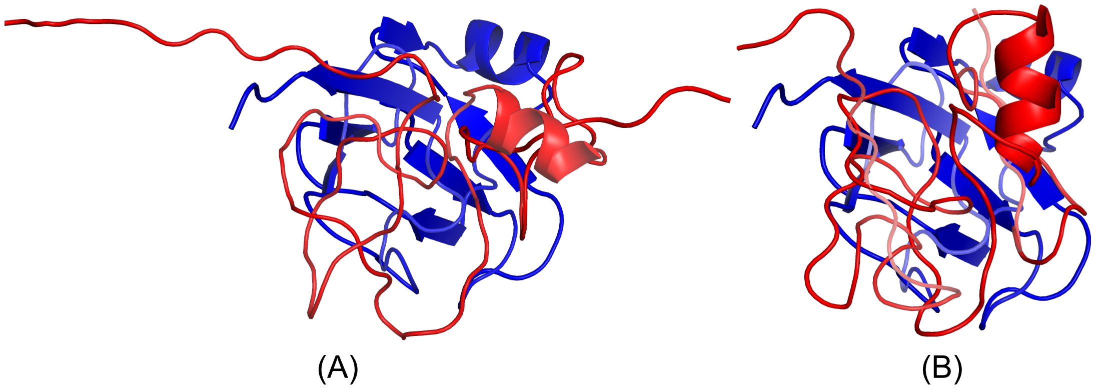

Figure 14: Predicted structure for protein `1npsA` by using CONFOLD (A) and CONFOLD+ISSEC (B). Here the predicted structures are shown in red whereas the native structure is shown in blue.

[1] Andreani, J. and Söding, J. (2015). bbcontacts: prediction of β-strand pairing from direct coupling patterns. Bioinformatics, **31**(11), 1729–1737.

[2] He, K., Gkioxari, G., Dollár, P., and Girshick, R. (2017). Mask r-cnn. In Computer Vision (ICCV), 2017 IEEE International Conference on, pages 2980–2988. IEEE.

[3] McGuffin, L. J., Bryson, K., and Jones, D. T. (2000). The PSIPRED protein structure prediction server. Bioinformatics, **16**(4), 404–405.

[4] Ren, S., He, K., Girshick, R., and Sun, J. (2015). Faster r-cnn: Towards real-time object detection with region proposal networks. In Advances in neural information processing systems, pages 91–99.
